# Are Sarcopenia and Cognitive Dysfunction Comorbid after Stroke in the Context of Brain–Muscle Crosstalk?

**DOI:** 10.3390/biomedicines9020223

**Published:** 2021-02-22

**Authors:** Sophia X. Sui, Brenton Hordacre, Julie A. Pasco

**Affiliations:** 1 IMPACT—The Institute for Mental and Physical Health and Clinical Translation, School of Medicine, Barwon Health, Geelong, Deakin University, Victoria, VIC 3220, Australia; julie.pasco@deakin.edu.au; 2 IIMPACT in Health, Allied Health and Human Performance, University of South Australia, Adelaide, SA 5000, Australia; Brenton.Hordacre@unisa.edu.au; 3 Department of Medicine–Western Health, The University of Melbourne, St Albans, VIC 3021, Australia; 4 Barwon Health, University Hospital Geelong, Geelong, VIC 3220, Australia; 5 Department of Epidemiology and Preventive Medicine, Monash University, Prahran, VIC 3181, Australia

**Keywords:** stroke, post stroke, sarcopenia, cognitive dysfunction

## Abstract

Stroke is a leading cause of death and disability and is responsible for a significant economic burden. Sarcopenia and cognitive dysfunction are common consequences of stroke, but there is less awareness of the concurrency of these conditions. In addition, few reviews are available to guide clinicians and researchers on how to approach sarcopenia and cognitive dysfunction as comorbidities after stroke, including how to assess and manage them and implement interventions to improve health outcomes. This review synthesises current knowledge about the relationship between post-stroke sarcopenia and cognitive dysfunction, including the physiological pathways, assessment tools, and interventions involved.

## 1. Introduction

Stroke is an episode of acute neurological dysfunction caused by the interruption of blood supply to the brain, leading to the death of brain cells. Medical interventions for stroke that have been successful in animal models have had poor efficacy in clinical studies. The complexity and heterogeneity of human diseases and related comorbidities may partly explain this, which may render neuroprotective drugs ineffective in clinical practice [1]. Stroke is linked to vascular dementia, which can be caused by chronic reduced blood flow to the brain [2] or may result from acute strategically located lesions, such as in the thalamus or inferior parietal lobule. The possible underlying biological mechanisms linking stroke and vascular dementia include oxidative stress, inflammation, and alterations to microribonucleic acid and brain proteins [2,3,4,5]. In addition, ageing and comorbidities have been recognised as major risk factors related to this link [1,5]. However, not all people who suffer a stroke are likely to develop vascular dementia. Understanding the developing trajectory between the two conditions and identifying biological mechanisms that link stroke events and the development of vascular dementia would enable the recommendation of lifestyle changes and/or novel interventions for cognitive reserve and flexibility. Post-stroke cognitive dysfunction—that is, diminished or impaired mental and/or intellectual function after stroke [6]—could be ameliorated by early intervention.

Another adverse health outcome of stroke is post-stroke sarcopenia, defined as the loss of muscle mass and function after a stroke [7]. Cognitive dysfunction and sarcopenia appear to be reported simultaneously in patients with stroke [8], indicating the likely parallel progression of comorbid sarcopenia and cognitive decline; however, the links between post-stroke sarcopenia and post-stroke cognitive dysfunction and its underlying mechanisms have rarely been investigated simultaneously. In Australia, the Geelong Osteoporosis Study reported that markers of sarcopenia (slow gait speed, poor handgrip strength, and low muscle density) were negatively associated with performance in some domains of cognitive function, including psychomotor function, visual learning, attention, and overall cognitive function in a general geriatric population [9,10]. These findings align with those of other studies on the links between sarcopenia and cognitive dysfunction in ageing [11,12]. Given the emerging evidence that links sarcopenia with cognitive dysfunction, it is possible that they share common mechanisms or underlying physiology [13]. If there are clear links between sarcopenia and cognitive dysfunction after stroke, the co-design of interventions for post-stroke sarcopenia and cognitive dysfunction may be achievable and cost-effective and produce greater clinical gain [14]. 

This review presents a critical appraisal of the scientific literature on the association between post-stroke sarcopenia and cognitive dysfunction and their underlying mechanisms. It describes the current assessment tools, summarises up-to-date interventions, identifies future directions, and finally proposes novel screening tools for use in population-based studies. 

## 2. Post-Stroke Cognitive Dysfunction

Cognitive dysfunction is more common in people with stroke compared to healthy age-matched adults. For example, an Irish cross-sectional study [15] examined the odds of nursing home residents who had experienced a stroke having cognitive dysfunction compared to residents without stroke at the time of admission. Among 643 residents, the prevalence of cognitive dysfunction and dementia was significantly higher in those who had experienced a stroke before admission. Further, post-stroke cognitive dysfunction was a predictor of negative physical and mental health outcomes, such as mortality, dependency, mood disorders, and hospitalisations [16], and an independent indicator of stroke recurrence [17]. Fitri et al. [18] found that of 38 patients (39.5% women) after stroke, 17 (44.7%) were cognitively impaired. Cognitive function was assessed using the Mini Mental State Examination (MMSE), forward digit span and backward digit span tests. 

A Norwegian longitudinal study of 617 patients with stroke (mean age 72 ± 12, 42% women) investigated the effect of stroke severity on cognitive dysfunction [19]. The assessed domains of cognitive function included attention, executive function, memory, language, and perceptual-motor function. Cognitive function was assessed at three months and/or 18 months after stroke. The severity of cognitive dysfunction and stroke were determined by the Diagnostic and Statistical Manual of Mental Disorders DSM-5 criteria and the National Institutes of Health Stroke Scale (NIHSS), respectively. This study found that executive function and language improved over the stroke recovery period; post-stroke cognitive dysfunction was common for all stroke subtypes at the two follow-up points. Even a minor stroke appeared to be a risk factor for developing cognitive dysfunction. A longitudinal study in Norway [20] included 324 patients with minor stroke followed at 3-month and 12-month intervals (37 patients were lost to follow-up). The authors observed cognitive improvement during the follow-up period, but the prevalence of cognitive dysfunction remained high (29.5%) 12 months after stroke. 

Prevalence estimates of post-stroke cognitive dysfunction vary between studies, depending on the definitions, subtypes, and severity of the stroke; the length of follow-up periods; and the cognition assessment tools used [21,22]. A systematic review reported that the prevalence of cognitive dysfunction was 14–88% among 11 studies for post-intracerebral haemorrhage, which is typically associated with a higher prevalence of dementia than ischaemic stroke [23]. Together, these studies emphasise the magnitude and significance of cognitive impairment following stroke. The studies included in this section are summarised in Table 1.

## 3. Post-Stroke Sarcopenia or Cachexia

Cachexia is a condition associated with general ill-health, malnutrition, extreme weight loss, and muscle wasting and is a symptom of many chronic conditions [24]. Meanwhile, sarcopenia is often described as age-related muscle mass and functional decline [25]. In the stroke literature, the terms sarcopenia and cachexia appear to be employed interchangeably, possibly because researchers from different disciplines have a different understanding of these conditions. In general, sarcopenia is more accurately defined as age-based skeletal muscle decline, while cachexia is diseases-based [24]. Recently, there have been suggestions that low muscle strength, rather than low muscle mass, be considered the major determinant of sarcopenia [25]. Thus, to further investigate whether the loss of muscle mass and function is disease-based or age-related, we suggest that sarcopenia and cachexia are not regarded as interchangeable. 

Scherbakov et al. [26] investigated changes in body composition and body weight after ischemic stroke and their association with functional outcomes. They studied 67 consecutive patients (42% women, mean age 69 years, mean body mass index 27.0) with acute ischemic stroke and mild to moderate neurological deficit assessed using the NIHSS. Assessment data were obtained during the acute phase (4 ± 2 days) and at the 12-month follow-up. Body composition was examined by dual-energy X-ray absorptiometry (DXA). Body weight loss ≥5% within one year and additional clinical signs were considered to denote cachexia. In this cohort study, one in five patients with ischemic stroke developed cachexia within 12 months of s stroke. Cachexia was associated with the lowest functional and physical capacity.

There is no universally accepted operational definition of sarcopenia [27,28], meaning that the prevalence estimates of post-stroke sarcopenia vary. A study in the United States of America (USA) [29] aimed to determine the prevalence of sarcopenia in post-stroke survivors using multiple methods and reported a prevalence of 14–18%. Su et al.’s systematic review and meta-analysis [30] reported that the pooled prevalence estimate of post-stroke sarcopenia was 42% (95%CI: 33–52%, I^2^ = 91%) in patients with stroke. This review included seven studies, six from Asian countries and one from the USA. The authors concluded that longitudinal studies are needed to establish the incidence estimates of sarcopenia after stroke.

Skeletal muscle mass alone seems to be an indicator of health outcomes after stroke. Ohyama et al. [31] examined the association between lean mass and clinical outcomes of acute ischemic stroke (severe neurological impairment and functional status on admission). Of 164 Japanese patients (34% women), 101 (62%) were identified as having lean mass deficits, determined by bioelectrical impedance analysis (BIA). This study found that lean mass deficits were not only associated with a worse status of conditions (i.e., severe neurological impairment and functional status) at admission but with poor functional outcomes and longer durations of hospital stay.

Consistent with the above, Nagano et al. [32] suggested that preserving muscle mass is associated with better functional recovery in post-stroke patients with sarcopenia. This retrospective study included 272 patients (44% with sarcopenia) recruited from hospitals in Japan. Sarcopenia was assessed as the loss of lean mass plus decreased handgrip strength, according to the Asian Working Group for Sarcopenia’s (AWGS) definition [33]. The Functional Independence Measure (FIM) was used to assess stroke recovery. The authors suggested that exercise and nutritional interventions were needed to reduce sarcopenia and achieve better stroke recovery. Matsushita et al. [34] examined the relationship between sarcopenia (using the European Working Group on Sarcopenia in Older People’s definition [35]) and ability to perform daily living activities (assessed using the FIM motor domain score [36]) in post-stroke patients after rehabilitation intervention. This study involved 267 participants (56% men), 129 with sarcopenia. The results suggested that sarcopenia independently predicts the inability to engage in daily living activities after rehabilitation for men; a potential sex interaction in this relationship means that further investigation is needed.

Concurrent sarcopenia and excessive fat accumulation is known as sarcopenic obesity and is more common with increasing age and adiposity [37]. Matsushita et al. revealed that sarcopenic obesity was independently associated with lower ability to perform activities of daily living in post stroke-patients undergoing a convalescent rehabilitation program [38]. In this study, sarcopenia was defined using handgrip strength and the AWGS’s muscle mass index, while obesity was defined as body fat mass index. Of 376 patients (mean age 78 years), 17% were obese, 32% had sarcopenia, and 28% had sarcopenic obesity. Sarcopenia obesity was independently associated with lower activities of daily living capability; however, obesity or sarcopenia alone were not. The studies included in this section are summarised in Table 1.

## 4. Are Post-Stroke Sarcopenia and Cognitive Dysfunction Comorbid?

Evidence emerging from observational and experimental studies has shown that human body composition and brain function are linked [9,10,13,39,40,41,42,43]. Although sarcopenia and cognitive dysfunction are often considered separate multidimensional concepts, they may have common risk factors and biological pathways. In general, advanced age contributes to the decline of skeletal muscle health and cognitive function [9,10]. In older patients in hospital settings, sarcopenia and cognitive dysfunction are strongly associated [44]. Complex genetic and modifiable factors, such as decreased blood supply, reduced social engagement, lack of exercise, malnutrition, and reduced neurological performance interplay in this relationship during ageing [45]. Recently, the concept of muscle–cognition crosstalk has received substantial attention in the literature [46]; however, stroke-related sarcopenia and cognitive dysfunction are rarely discussed as comorbidities. We acknowledge the complexity of discussing the muscle–cognition relationship in a disease context; disease may exacerbate the natural effects of ageing and accelerate the co-development of sarcopenia and cognitive dysfunction.

The current literature contains little epidemiological evidence about the overlap of post-stroke sarcopenia and cognitive dysfunction. The lack of consensus about definitions, assessment tools and techniques in stroke-related cognitive dysfunction and sarcopenia further reduce the quality of the evidence. Given the high prevalence of the two conditions in population-based studies and in hospitalised older patients [44], studies are needed to determine whether sarcopenia and cognitive dysfunction can be considered comorbidities and associated by definition in stroke populations.

## 5. What Are the Potential Underlying Biological Mechanisms If Post-Stroke Sarcopenia and Cognitive Dysfunction Are Comorbid?

Sui et al have identified a potential role of inflammation linking muscle density and visual learning and memory in a population-based study [10]; in stroke literature, the role of inflammation markers is less clear and inconsistent. A recent review highlighted that changes in molecular biomarkers such as C-reactive protein (CRP), interleukin 6 (IL-6) and IL-10 in blood, urine, and other body fluids are associated with post-stroke cognitive decline [47]. Yoshimura et al.’s [48] retrospective cohort study investigated the association between systemic inflammation, post-stroke sarcopenia, and the functional consequences (motor, cognitive function, and overall) in post-stroke patients. The study included 204 Japanese patients (47% women, mean age 74.1 years) who were admitted to rehabilitation wards between 2015 and 2017. A modified Glasgow Prognostic Score, determined by the serum levels of CRP and albumin, was adopted to evaluate systemic inflammation (participants who had acute or chronic high-grade inflammatory diseases were excluded). Sarcopenia was identified using the AWGS definition, and the outcome was assessed using the FIM. The authors identified 39.7% patients as having sarcopenia and reported that inflammation was independently associated with sarcopenia and negative motor outcomes. However, sarcopenia was not associated with cognitive dysfunction after adjusting for confounders. For future research, Yoshimura et al. recommended models that include multiple paths of influence and interactions, suggesting that inflammation cannot be excluded as a potential mechanism linking sarcopenia and cognitive impairment after stroke.

Brain-derived neurotrophic factor (BDNF), a neurotrophin growth factor that is important for nervous system development and the survival of existing neurons, is considered a protective factor for stroke. An integrative review has reported lower BDNF levels in patients with stroke [49]; in one data-driven study, stroke patients with low levels of serum BDNF had significantly higher prevalence of cognitive dysfunction than healthy controls. Another study found that BDNF levels increased during motor rehabilitation following ischemic stroke [50]. A randomised controlled trial (RCT) in Taiwan compared the effect of high-intensity interval training (HIIT) and moderate-intensity continuous training on serum BDNF and other outcomes in stroke patients. This study found that HIIT was more effective, because increased BDNF promoted neuronal activity and induced better health outcomes. Future studies that focus on neurologic recovery using intensity-dependent exercise should consider BDNF as a marker [51].

## 6. What Assessment Tools Are Available for Post-Stroke Sarcopenia?

The standard techniques for assessing body composition—DXA and peripheral quantitative computed tomography (pQCT) (detailed in the following section)—may also be applied in the stroke population. However, in some circumstances they may not be suitable as many assessment tool for muscle mass and volumes are sensitive to movement (e.g., pQCT and DXA), and people suffering from stroke may not be able to perform physical performance (e.g., walking and HGS testing). For example, Jung et al. [52] argued that an alternative assessment tool for post-stroke sarcopenia is needed, because the current suggested measurement tool (e.g., DXA) is age-related and limited to stroke-specific sarcopenia, especially for muscle performance and strength. Their study included 40 patients (56% women; mean age 67 years, SD = 15.4) who had experienced stroke but could walk independently. Muscle mass was assessed by measuring ultrasonographic muscle thickness [48] and by measuring skeletal lean mass using bioelectrical impedance analysis (BIA). Muscle strength was assessed by the Medical Research Council Sum-Score (obtained by calculating the muscle strength scores using manual muscle testing for the upper and lower limbs) [52], and muscle strength was also assessed by evaluate handgrip strength (obtained from a digital dynamometer). Physical performance was measured by the Berg Balance Scale [53] and by a 4-meter gait speed test. The correlations between each assessment for muscle mass, muscle strength, and muscle performance were examined and stroke severity, comorbidity, and nutritional status were considered as confounders. The results supported Jung et al.’s assertion that their proposed alternative measurements could be used to assess sarcopenia in stroke patients.

## 7. What Assessment Tools Are Available for Cognitive Dysfunction?

Montreal Cognitive Assessment (MoCA) is commonly used in the assessment of post-stroke cognitive function in patients [54]. In population studies, general cognitive dysfunction is commonly assessed using the MMSE or its modified versions. The MMSE is a valid and reliable tool and is used worldwide [55]. Recently, computer-based neuropsychological tests have been developed to evaluate cognitive function in the domains of memory, executive function, psychomotor function, information processing, and language [56].

The DSM-5 provides criteria for the diagnosis of neurocognitive disorders, including dementia and Alzheimer’s disease. Structured clinical interviews are used in combination with neuropsychology tests to identify individuals who may develop or already have dementia or Alzheimer’s disease for diagnostic and research purposes. Neuropsychological tests, together with neuroimaging techniques, have been used to identify early-stage Alzheimer’s disease in people with mild cognitive dysfunction [57]. Epidemiological studies are needed to obtain normative data in each cognitive domain for diagnostic purposes.

## 8. What Interventions Exist for Post-Stroke Rehabilitation, and What Are the Future Directions in the Context of Brain-Muscle Crosstalk?

Current post-stroke rehabilitation interventions treat muscle function (mainly motor function) and cognitive function separately. Irisawa et al.’s prospective study included 179 patients recruited from two stroke rehabilitation units in Japan [58]. Lean mass was assessed by BIA and nutrition status using the Geriatric Nutritional Risk Index. The capability of daily living (FIM scores) was measured at admission and four weeks later. The results showed that high skeletal muscle mass relative to body weight and better nutritional status was significantly associated with FIM scores at four weeks; no sex differences were identified. The authors suggested that muscle mass maintenance through nutritional management is essential for functional recovery in stroke patients. However, this study did not assess body composition changes during recovery or the evaluate muscle strength, so this omission provides some suggestion that the possible morbid characteristics of sarcopenia and cognitive dysfunction are not considered in the clinical settings.

Lathuiliere et al. [14] reviewed the evidence for nutritional and pharmacological interventions to prevent muscle wasting in stroke survivors. This review analysed eight RCTs and two cohort studies and could not identify an effective nutritional intervention; however, they noted that amino acid supplementation and anabolic steroid administration (separately) appeared to prevent a loss of muscle mass in post-stroke sarcopenia. Cognitive outcomes were not assessed in this study.

In a double-blinded RCT, Yoshimura et al. [59] examined whether a leucine-enriched amino acid supplement could improve sarcopenia conditions (muscle weakness and loss of muscle mass) in post-stroke patients over eight weeks. The study involved 44 post-stroke older patients (21 in the intervention group and 23 in the control group) with sarcopenia (defined using AWGS criteria). Both groups undertook resistance training and a post-stroke rehabilitation program. Outcomes measures, including physical function, appendicular muscle mass, and muscle strength, were measured at baseline and after the intervention. The leucine-enriched amino acid supplementation was shown to reduce sarcopenia.

Ihle-Hansen et al.’s [60] 18-month RCT examined the effects of individualized regular coaching and exercise on post-stroke cognition. The Trail Making Test A and B and MMSE were used to assess cognition, with data collected three and 21 months after stroke. Of 362 patients aged 72 years (39.5% women), 177 patients were in the intervention group and 185 were in the control group (usual care). This study did not detect a clinically significant difference in cognition between the two groups; however, increased adherence to the intervention was associated with improved cognition, suggesting an interaction between cognitive function improvement and motivation. The authors suggested incorporating cognitive interventions into cardiac rehabilitation.

Dysphagia (difficulty in swallowing) is considered to be associated with stroke-induced sarcopenia; however, a retrospective cohort study in Japan [61] revealed a positive effect of whole-body exercise on clinical outcomes including dysphagia in stroke patients undergoing rehabilitation. This study included 637 patients (51% women, 23% with dysphagia) with a mean age of 73 years. Dysphagia was assessed using the Food Intake Level Scale. The intervention included a chair stand exercise (median daily frequency = 36) in addition to the convalescent rehabilitation program. The results showed that chair-stand exercise was independently associated with reduced dysphagia.

## 9. Is Population Screening for Sarcopenia and Cognitive Dysfunction Needed to Prevent Stroke?

### 9.1. Pre-Stroke Sarcopenia

In previous sections, we demonstrated that body composition alterations, including sarcopenia, are a consequence of stroke. Interestingly, sarcopenia may also be a risk factor for the onset of stroke. Minn and Suk [62] conducted a community-based cross-sectional study involving 722 participants (age range 50–75 years) without stroke and dementia; based on brain and muscle scans, they concluded that increased lean mass may protect against brain structure changes and against ischemic stroke, particularly in men. Nozoe et al. [63] investigated the association between sarcopenia and the severity of stroke. Pre-stroke sarcopenia was assessed using a questionnaire (SARC-F) and stroke severity using the NIHSS. In 183 patients (44% women, 15% with pre-stroke sarcopenia), pre-stroke sarcopenia was a predictor of severe stroke before and after adjusting for confounders. A prospective cohort study [64] by the same research group investigated the association between pre-stroke sarcopenia (determined by SARC-F) and functional outcomes after stroke (using a modified Rankin Scale score) at three months in patients with acute stroke. This study involved 152 patients (47% women, 18 with pre-stroke sarcopenia) with a mean age of 76 years. Patients with pre-stroke sarcopenia were seven times more likely to develop poor health outcomes at the assessment endpoint.

A South Korean retrospective cohort study [65] examined the association between existing sarcopenia (assessed within two weeks of hospitalisation) at the onset of stroke and functional recovery six months after stroke. This study included 194 hemiplegic post-stroke patients. The results revealed that patients with pre-existing sarcopenia were almost three times more likely to have poor clinical health outcomes at six months. Additionally, they reported a sex-specific difference, in that women were more than twice as likely to develop poor outcomes as men.

Note that the mechanisms linking pre-stroke sarcopenia and clinical stroke outcomes are unclear. One reason for this could be reduced blood flow. The presence of sarcopenia, lack of physical activity, and poor nutrition are independent risk factors for both stroke and sarcopenia. This could reflect the fact it is more difficult to rehabilitate someone with sarcopenia because of pre-existing muscle weakness, rather than sarcopenia being a specific risk factor for more severe stroke.

Population-based studies help identify risk factors and mechanisms before the onset of certain diseases. Modifiable risk factors of stroke, such as hypertension, diabetes mellitus, obesity, tobacco smoking, hyperlipidaemia, poor diet/nutrition, and physical inactivity, [66] are considered in large-scale cohort studies. Both sarcopenia and cognitive dysfunction among older adults can be screened at the population level. Therefore, coexisting sarcopenia and cognitive dysfunction are assessable in a population-based study, enabling stroke to be predicted and the underlying pathological pathways to be understood. The studies included in this session are summarised in Table 1.

### 9.2. Common Techniques for Assessing Sarcopenia in Population-Based Studies

Lean mass can be obtained from whole-body scans using DXA, a rapid, accurate, and non-invasive method for measuring body composition including bone, fat, and lean tissue. Lean tissue assessed by this technology comprises non-fat and non-bone tissue and correlates well with skeletal muscle mass quantified by magnetic resonance imaging [67].

Muscle mass and density can be measured by pQCT at the tibial shaft and proximal forearm sites. In the GOS [10], standard transverse scans were performed at 4% and 66% of the radial and tibial length. Radial and tibial data were obtained and analysed separately. Density thresholds were used to distinguish bones, muscle, and fat tissue (fat: <15 mg/mm^3^; muscle: 15–180 mg/mm^3^; bone: >180 mg/mm^3^) [68].

Magnetic resonance imaging (MRI) has the best image resolution and a high level of accuracy and is central to much research in brain-imaging studies. While MRI does not use X-rays or other ionising radiation, it is expensive and not widely available [69].

A more direct measure of muscle mass uses bioelectric impedance analysis (BIA) to determine total body water, lean mass, and body fat. BIA detects impedance to the flow of a small current (approximately 1–10 μA), usually measured between the wrist and opposite ankle via two or four electrodes that make contact with the skin. While BIA is an inexpensive tool that is portable and rapid, it has limited accuracy [69,70,71]

Handgrip strength (kg) can be measured using hand-held dynamometry [72]. The testing procedure should be explained and demonstrated to each participant before measurement. With the participant seated in a comfortable position and the arm holding the dynamometer flexed at the elbow to 90 degrees, the participant is asked to squeeze the device as hard as possible for several seconds and the peak reading is recorded.

Leg muscle strength can be assessed using a break test technique to measure peak eccentric muscle strength in the legs using a hand-held dynamometer that is a reliable method for assessing lower-limb muscle strength in adults, and the population-based normative data for Caucasians are available to clinically identify people who are at low levels of leg muscle strength [73].

Usual gait speed (shoes on) is typically determined by measuring how many seconds the participant takes to walk a distance of four metres and is recorded as the walking speed (m/s) [9].

Another test of physical performance is “the timed-up-and-go (TUG) test”, where the time taken to stand up from a chair and walk to a marked line (usually over a distance of 3 metres) then return and sit down again is recorded. This test involves both walking speed and balance [74].

The short physical performance battery (SPPB) is a validated tool that evaluates lower extremity physical performance using three tests: standing balance, walking speed, and chair stand tests [75]. The score ranges from 0 to 12 (worst to best performance) by combining the three times for the subtests [75].

### 9.3. Functional Near-Infrared Spectroscopy

Functional Near-Infrared Spectroscopy (fNIRS) is a neuroimaging technique that is non-invasive, cost-effective, safe, rapid to set up, portable, and non-motion-sensitive [76]. Due to these advantages, fNIRS has been widely used in studies of particular clinical populations, such as Alzheimer’s disease, depression, and other chronic neurological disorders [77]. fNIRS maps neuronal activity by applying near-infrared spectrum light that can travel through tissue. It is sensitive to brain neurovascular changes during neuroactivity, as the secondary measurement for neuro-connectivity localisation changes through detecting subtle brain blood flow changes (not measuring neuronal activities directly) [78]. To be more specific, near-infrared light is absorbed by oxygenated haemoglobin and deoxygenated haemoglobin in the cortex. An increase in oxygenated haemoglobin is considered an indicator of neuronal activity, and a canonical hemodynamic response involves an increase in oxygenated haemoglobin accompanied by a decrease in deoxygenated haemoglobin [78].

Butler et al. noted that fNIRS has been used widely to investigate brain activities in response to transcranial magnetic stimulation in patients with stroke, and recommended using fNIRS for speech and language studies in stroke [79]. Another literature review examined how fNIRS may be applied to the study of gait disorders in stroke and discussed its application for assessing rehabilitation programs [80]. Most studies in this field are case-control studies in clinical populations with a low statistical power; no longitudinal observational studies have addressed risk factors for stroke in the context of concurrent post-stroke sarcopenia and cognitive impairment.

## 10. Conclusions

The literature to date contains little evidence about the links between post-stroke sarcopenia and cognitive function. Post-stroke sarcopenia and cognitive dysfunction may be comorbid and associated as they often occur simultaneously and share some similar underlying mechanisms. A future direction for stroke rehabilitation research might be to involve testing combinations of interventions for cognitive function and physical performance.

## Figures and Tables

**Table 1:** Summary of key studies included in the literature review.

Author, Year; Country/Region	Study Type/Follow-Up Period	Aim	Participant Characteristics	Results (Key Findings)
**Post-stroke cognitive dysfunction**				
Donnelly et al. 2020 [15]; Irish	Cross-sectional survey;	Investigate the odds of nursing home residents who had experienced stroke having cognitive dysfunction compared to residents without stroke at the time of admission.	643 residents in 13 randomly selected nursing homes	Prevalence of cognitive dysfunction and dementia were significantly higher in those who had experienced stroke before admission.
Fitri et al. 2020 [18]; Indonesia	Cross-sectional;	Determine the impact of working memory impairment after stroke on activities of daily living.	38 patients, 23 (60.5%) males; the main age was 58.8 ± 10.38 years	Of 38 patients (39.5% women) after stroke, 17 (44.7%) were cognitively impaired.
Aam et al. 2020 [19]; Norway	Longitudinal; three months and/or 18 months after stroke	Investigate the role of the significance of time and etiologic stroke subtype for the probability of Post-stroke cognitive impairment, severity, and cognitive profile.	617 patients with stroke (mean age 72 ± 12, 42% women)	Executive function and language improved over the stroke recovery period; post-stroke cognitive dysfunction was common for all stroke subtypes at the two follow-up points. Even a minor stroke appeared to be a risk factor for developing cognitive dysfunction.
Morsund et al. 2020 [20]; Norway	Longitudinal; followed at 3-month and 12-month intervals	Investigate the minor stroke effect on developing of cognitive and emotional symptoms	324 patients (120 female) with minor stroke; the main age was 58.0 ± 10.0 years	The authors observed cognitive improvement during the follow-up period, but the prevalence of cognitive dysfunction remained high (29.5%) 12 months after stroke.
Donnellan and Werring. 2020 [23]. Ireland	Systematic review	Identify and quantify studies that focused on cognitive dysfunction before and after Intracerebral haemorrhage.	Nineteen studies were included	Prevalence of cognitive dysfunction was 14–88% among 11 studies for post-intracerebral haemorrhage, which is typically associated with a higher prevalence of dementia than ischaemic stroke.
**Post-stroke sarcopenia or cachexia**				
Scherbakov et al. 2019 [26]; Germany	Longitudinal study; assessed at the acute phase (4 ± 2 days) and at 12-month follow-up.	Investigated changes of body composition and body weight after ischemic stroke and their association with functional outcomes.	67 consecutive patients (42% women, mean age 69 years, mean body mass index 27.0 kg/m^2^) with acute ischemic stroke and mild to moderate neurological deficit.	One in five patients with ischemic stroke developed cachexia within 12 months of stroke. Cachexia was associated with the lowest functional and physical capacity.
Ryan et al. 2017 [29]; United States of America (USA)	Case-control	Determine the prevalence of sarcopenia in post-stroke survivors using multiple methods.	190 participants (61% men; aged 40 to 84 years) with mild to moderately disabled >6 months after onset of stroke.	Reported sarcopenia prevalence of 14–18%.
Su et al.2020 [30]; Japan	Systematic review and meta-analysis	Search the prevalence of sarcopenia in stroke survivors.	This review included seven studies, six from Asian countries and one in the USA.	The pooled prevalence estimate of post-stroke sarcopenia was 42% (95%CI: 33–52%, I^2^ = 91%) in patients with stroke.
Ohyama et al. 2020 [31]; Japan	Cross-sectional	Examined the association between lean mass and clinical outcomes of acute ischemic stroke (severe neurological impairment and functional status on admission).	164 geriatric patients with acute ischemic stroke (108 males).	Of 164 Japanese patients (34% women), 101 (62%) were identified as having lean mass deficits; this study found that lean mass deficits were not only associated with worse status of conditions (i.e., severe neurological impairment and functional status) at admission but with poor functional outcomes and longer duration of hospital stay.
Nagano et al. 2020 [32]; Japan	Retrospective cohort study	Investigate the relationship between changes in skeletal muscle mass and functional outcomes in patients with sarcopenia after stroke.	272 patients (mean age 79 years, 70 females; 44% with sarcopenia) recruited from hospitals.	Preserving muscle mass is associated with better functional recovery in post-stroke patients with sarcopenia.
Matsushita et al. 2020 [34]; Japan	Cross-sectional	Examined the relationship between sarcopenia and ability to perform daily living activities in post-stroke patients after rehabilitation intervention.	267 participants (56% men), 129 with sarcopenia.	Sarcopenia independently predicts the inability to engage in daily living activities after rehabilitation for men; a potential sex interaction in this relationship means further investigation is needed.
Matsushita et al. 2020 [38]; Japan	Cross-sectional	Revealed that sarcopenic obesity was independently associated with lower ability to perform activities of daily living in post stroke-patients undergoing a convalescent rehabilitation program.	Of 376 patients (mean age 78 years), 17% were obese, 32% had sarcopenia and 28% had sarcopenic obesity.	Sarcopenia obesity was independently associated with lower activities of daily living capability; however, obesity or sarcopenia alone were not.
**Pre-stroke sarcopenia or cachexia**				
Minn and Suk 2017 [62]; Japan	Cross-sectional	Investigate whether increasing muscle mass can prevent stroke.	722 participants (age range 50–75 years) without stroke and dementia.	Increased lean mass may protect against brain structure changes and against ischemic stroke, particularly in men.
Nozoe et al. 2019; Japan [63]	Cross-sectional	Investigated the association between sarcopenia and the severity of stroke.	In 183 patients (44% women, aged ≥ 65 years; 15% with pre-stroke sarcopenia).	Pre-stroke sarcopenia was a predictor of severe stroke before and after adjusting for confounders.
Nozoe et al. 2019 [64]; Japan	Prospective cohort study	Investigated the association between pre-stroke sarcopenia and functional outcomes after stroke at three months in patients with acute stroke.	This study involved 152 patients (47% women, 18 with pre-stroke sarcopenia) with a mean age of 76 years.	Patients with pre-stroke sarcopenia were seven times more likely to develop poor health outcomes at the assessment endpoint.
Jang et al. 2020 [65]; South Korean	Retrospective cohort study	Examined the association between existing sarcopenia (assessed within two weeks of hospitalisation) at onset of stroke and functional recovery six months after stroke.	This study included 194 hemiplegic post-stroke patients.	The results revealed that patients with pre-existing sarcopenia were almost three times more likely to have poor clinic health outcomes at six months. Additionally, they reported a sex-specific difference, in that women were more than twice as likely to develop poor outcomes as men.
